# Developing the IVIG biomimetic, Hexa-Fc, for drug and vaccine applications

**DOI:** 10.1038/srep09526

**Published:** 2015-04-27

**Authors:** Daniel M. Czajkowsky, Jan Terje Andersen, Anja Fuchs, Timothy J. Wilson, David Mekhaiel, Marco Colonna, Jianfeng He, Zhifeng Shao, Daniel A. Mitchell, Gang Wu, Anne Dell, Stuart Haslam, Katy A. Lloyd, Shona C. Moore, Inger Sandlie, Patricia A. Blundell, Richard J. Pleass

**Affiliations:** 1 Bio-ID Center, School of Biomedical Engineering, Shanghai Jiao Tong University, Shanghai, 200240 P. R. China; 2 Centre for Immune Regulation (CIR) and Department of Immunology, Oslo University Hospital Rikshospitalet, P.O. Box 4956, Oslo N-0424, Norway; 3 Department of Pathology and Immunology, Washington University School of Medicine, St. Louis, MO 63110, USA; 4 Liverpool School of Tropical Medicine, Pembroke Place, Liverpool, L3 5QA, UK; 5 Clinical Sciences Research Laboratories, Warwick Medical School, University of Warwick, Coventry CV2 2DX, UK; 6 Department of Life Sciences, Imperial College London, South Kensington Campus, London SW7; 7 CIR and Department of Biosciences, University of Oslo, N-0316 Oslo, Norway

## Abstract

The remarkable clinical success of Fc-fusion proteins has driven intense investigation for even more potent replacements. Using quality-by-design (QbD) approaches, we generated hexameric-Fc (hexa-Fc), a ~20 nm oligomeric Fc-based scaffold that we here show binds low-affinity inhibitory receptors (FcRL5, FcγRIIb, and DC-SIGN) with high avidity and specificity, whilst eliminating significant clinical limitations of monomeric Fc-fusions for vaccine and/or cancer therapies, in particular their poor ability to activate complement. Mass spectroscopy of hexa-Fc reveals high-mannose, low-sialic acid content, suggesting that interactions with these receptors are influenced by the mannose-containing Fc. Molecular dynamics (MD) simulations provides insight into the mechanisms of hexa-Fc interaction with these receptors and reveals an unexpected orientation of high-mannose glycans on the human Fc that provides greater accessibility to potential binding partners. Finally, we show that this biosynthetic nanoparticle can be engineered to enhance interactions with the human neonatal Fc receptor (FcRn) without loss of the oligomeric structure, a crucial modification for these molecules in therapy and/or vaccine strategies where a long plasma half-life is critical.

Fc-fusion proteins are a well-established class of therapeutics^1^, in fact presently exhibiting the greatest growth rate of all biologics in the United States^2^. Notwithstanding this success though, there is great interest in identifying novel approaches to improve their efficacy and safety while expanding their range of potential clinical applications to other areas such as vaccines^3^ and replacements for intravenous immunoglobulin (IVIG) therapy^1^,^4^. However, one well-recognized drawback of the present Fc-fusion design for many of its potentially new applications is its monomeric structure: it is not able to cross-link multiple receptors with the high affinity required for enhanced function.

In particular, several diseases are known to be regulated by the activity of low-affinity inhibitory Fc receptors, including those on the surface of human B cells, such as FcγRIIb^5^ and FcRL5^6^ and those on macrophages and dendritic cell (DC) surfaces, such as FcγRIIb^7^ and dendritic cell-specific intercellular adhesion molecule-3-grabbing non-integrin (DC-SIGN)^8^,^9^. Of note, DC-SIGN is a C-type lectin and indeed there is a strict requirement of glycosylation for its association with IgG^9^,^10^. In particular, a number of studies have implicated α2,6-sialylation of the Fc-glycans as critically important for this interaction with DC-SIGN, although there is recently a great deal of debate on this issue^10^,^11^,^12^,^13^.

Intriguingly, each of these receptors is also targeted by pathogens in their attempt to inhibit immune responses involved in their removal^14^,^15^,^16^. Taken together, FcγRIIb, FcRL5, and DC-SIGN may thus limit immune cell activation against chronic pathogens or self-reactive antigen, and approaches that have the potential to target these receptors with high affinity/avidity may prove beneficial in therapies, including IVIG, aimed at controlling pro-inflammatory disease^1^,^4^.

We also note that the monomeric structure of present Fc-fusions also prevents their interaction with complement^17^,^18^, which significantly limits their application in cancer therapies where complement activation may be desirable^19^. Multimerization would also be expected to significantly enhance their interaction with the salvage neonatal Fc-receptor (FcRn), a crucial association that significantly prolongs the plasma half-life and likewise therapeutic and/or vaccine activity of any Fc-containing protein^1^,^20^.

We have recently developed an effective strategy to oligomerize monomeric Fc into well-defined hexameric oligomers (hexa-Fc) and demonstrated their binding to high-affinity Fc receptors^1^,^18^. Here we characterize the functional characteristics of this unique biosynthetic nanoparticle with several important immune effector systems, including low affinity B- and dendritic cell (DC) receptors, complement, and FcRn. We show that the binding to these effectors is strong, as expected from its oligomeric architecture, and thereby firmly establish this novel Fc nano-scaffold as an extremely promising alternative for future therapeutic and vaccine approaches.

## Results

### Binding of hexameric IgG1-Fc to human leucocytes

As a first step to evaluate the interaction of hexa-Fc (Figure S1) with human immune cells, we determined whether hexa-Fc binds to human circulating B cells and monocytes. In particular, CD19^+^ B cells from peripheral blood mononuclear cells of healthy human volunteers were screened by flow cytometry analysis (FACS). Despite high background binding of the anti-human IgG detecting reagent, most likely due to direct interactions with the IgG B cell receptor (BCR) and/or pre-bound IgG found on B cells, we could detect binding of hexa-Fc (Figure S2A). We also observed a robust association of hexa-Fc to CD14^+low^ and to a lesser extent to CD14^+high^ monocytes from these same individuals (Figure S2B). The increased binding of hexa-Fc to CD14^+^ monocytes may arise as a consequence of additional type 1 and type 2 FcγRs expressed by monocytes, including FcγRI, FcγRIIa, and FcγRIIIa, when compared to circulating CD19^+^ B cells that only constitutively express FcγRIIb and FcεRI^21^.

### FcRL5 and FcγRIIb are receptors for hexameric IgG1-Fc

**Figure 1:**
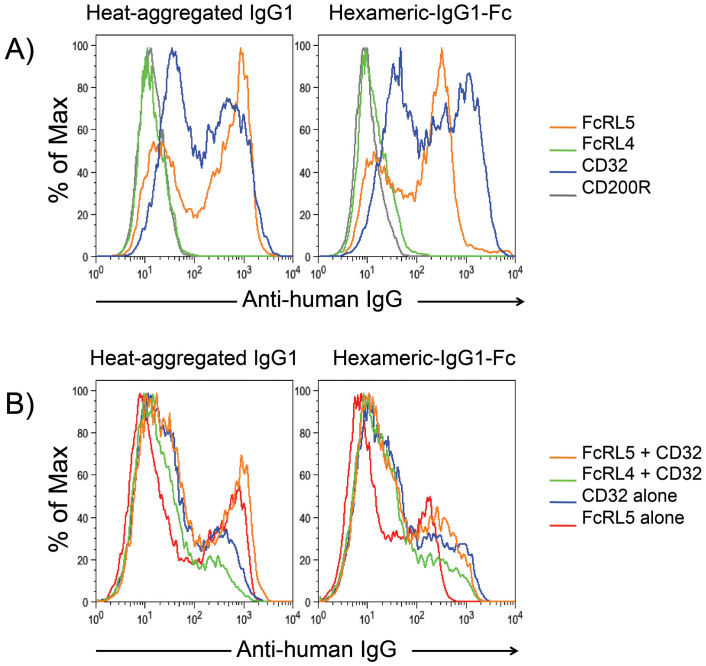
Hexa-Fc binds Fc-receptors with high avidity. (a) Hexa-Fc binds to FcRL5 and FcγRIIb (CD32b). Binding of either heat-aggregated IgG1 (left panel) or hexa-Fc (right panel) to cells expressing FcRL5 (orange trace), FcγRIIb (blue trace), CD200R control (grey trace) or FcRL4 control (green trace). Binding to CD200R and FcRL4 (human IgA receptor) are included as two negative controls. Data are representative of duplicate experiments. (b) Improved binding of hexa-Fc when FcγRIIb and FcRL5 are simultaneously expressed on the surface of 293 cells. Binding of heat-aggregated IgG1 (left panel) or hexa-Fc (right panel) to FcRL5/FcγRIIb double transfectants (orange trace), FcRL5 single transfectants (red trace), FcRL4/FcγRIIb double transfectants (green trace) and FcγRIIb single transfectants (blue trace). CD200 transfected controls are omitted from the overlays for clarity. Cell surface expression of receptors was confirmed using FITC-conjugated anti-FLAG M2 mAb or anti-FcγRIIb antibodies (as shown in Figure S3). Data represent duplicate experiments.

Human B cells are known to express two FcγRs for IgG, FcγRIIb and FcRL5^5^,^6^,^22^. To determine if these receptors could contribute to the interaction of hexa-Fc with B cells, and to overcome issues of background binding observed with isolated B cells, we evaluated the extent of binding of hexa-Fc to 293 cells transiently expressing these proteins or control CD200R and FcRL4 receptors by FACS^6^. We also studied the ability of heat-aggregated IgG to bind to the cells as a positive control and to provide some structural insight into the nature of these interactions. We found that both hexa-Fc and heat-aggregated IgG each bound significantly to the FcRL5- or FcγRIIb- expressing cells, whereas no binding was observed to cells expressing either control receptor (Figure 1A). We note that a more pronounced binding of hexa-Fc to FcγRIIb- than FcRL5-expressing cells was consistently observed, while the extent of expression of these receptors was the same (Figure 1C and Figure S3).

### Binding preferences of FcRL5 and FcγRIIb for hexa-Fc and heat-aggregated IgG

We hypothesized that simultaneous expression of both FcRL5 and FcγRIIb would lead to enhanced binding of heat-aggregated IgG or hexa-Fc. Although a marked improvement in binding of hexa-Fc was observed to the FcRL5/FcγRIIb double transfectants than to cells singly expressing FcRL5, the binding was no more than observed with FcγRIIb single- or FcRL4/FcγRIIb double-transfectants (Figure 1B). Two binding peaks were commonly observed for heat-aggregated IgG and/or hexa-Fc that most likely represent differences in receptor expression and/or differences in avidity of binding that arise from valence dependent interactions. The findings suggest that the binding of hexa-Fc to FcγRIIb was preferred over that to FcRL5. In contrast, heat-aggregated IgG bound to the transfectants in a predominantly FcRL5-dominated manner, as the binding to FcRL5/FcγRIIb double transfectants was comparable to that of cells singly expressing FcRL5 and greater than to the FcγRIIb single-transfectants. Further support of these receptor preferences is evidenced by the blocked binding of heat-aggregated IgG to cells first incubated with the anti-FcRL5 blocking mAb 509F6, whereas binding of hexa-Fc was less affected with this treatment (Figure S3).

### Interactions with FcRn

**Figure 2:**
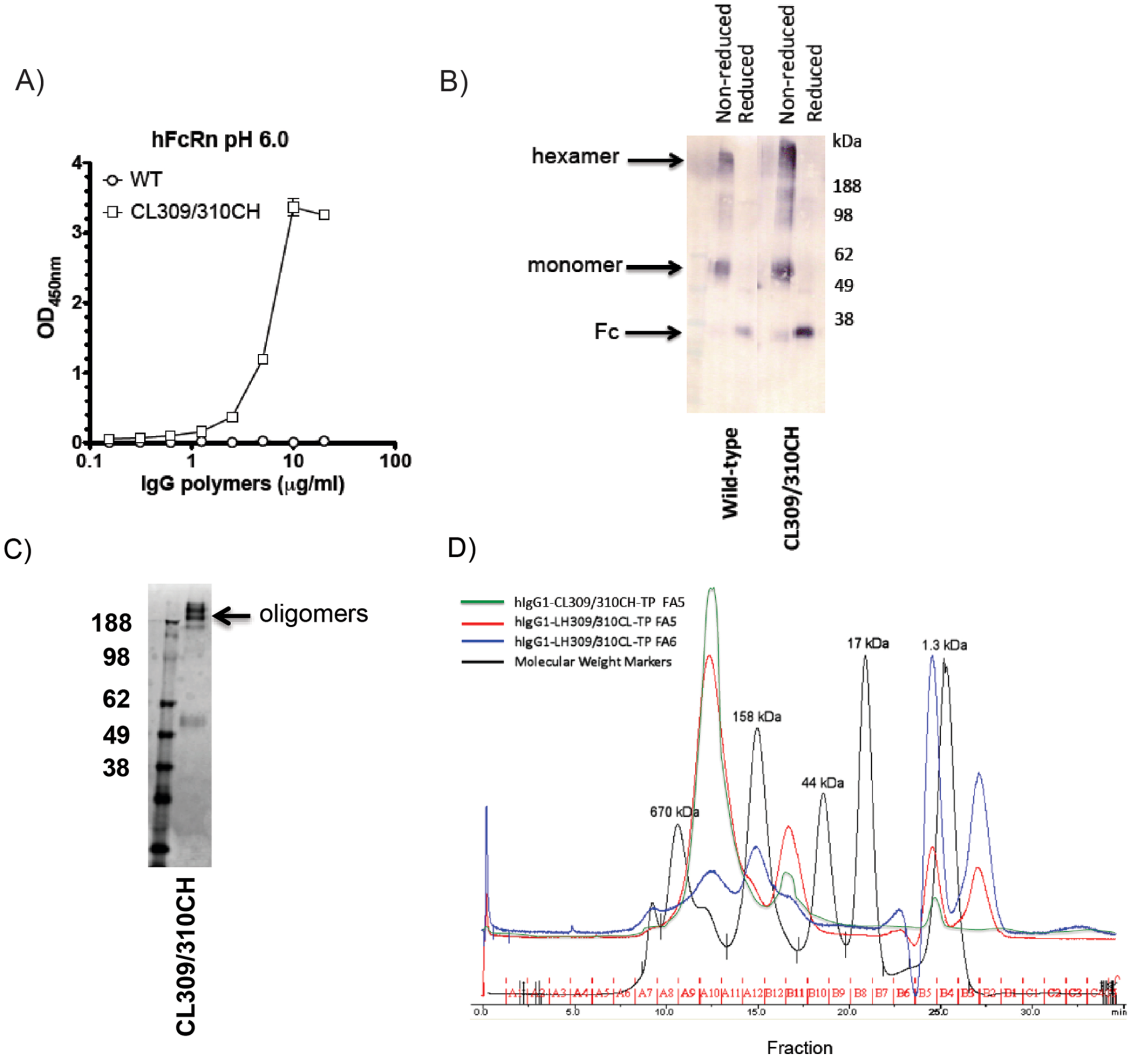
Mutant CL309/310CH forms higher order oligomers, including hexamers, and binds human FcRn. (a) Titrated amounts of hexa-Fc or the mutant CL309/310CH were coated onto wells of an ELISA plate. Binding of GST-fused human FcRn at pH6.0 as indicated was visualized using an HRP-conjugated anti-GST Ab. The values represent the average of triplicate determinations (±SD) from two independent experiments. (b) 5 μg of purified Fc and 5 μl SeeBlue2 pre-stained molecular weight markers were run under non-reducing or reducing conditions into 4–12% bis-Tris-acrylamide gradient gels and transferred to nitrocellulose. The human Fc was detected using a goat anti-human IgG conjugated to alkaline phosphatase. (c) 5 μg of the CL309/310CH mutant run under non-reducing conditions as in (b) and stained with Coomassie blue. (d) Size-exclusion chromatography (SEC) analysis on Superdex-200 10/300GL column showing the CL309/310CH mutant runs with an approximate molecular weight of 324 kDa (green trace). LH309/310CL control (red trace), irrelevant protein G fraction for LH309/310CL control (blue trace). Elution profiles of molecular weight standards are indicated by the black trace.

Hexa-Fc was previously shown not to bind human FcRn (and Figure 2A)^18^. The binding site for FcRn on IgG is localized at the Cγ2-Cγ3 junction and involves Ile253, His310, His433 and His435^23^,^24^. The pKa of histidine is 6.0–6.5 allowing the histidine residues to become protonated below physiological pH, enabling salt bridge formation with acidic residues on the FcRn, and explaining the strict pH dependence of IgG-FcRn interactions^25^. We reasoned that the lack of binding observed previously with hexa-Fc was due to the presence of leucine at 310 rather than a histidine, as found in IgG1-Fc. At the time we postulated that a histidine residue so close to the critical Cys309 might promote oxidation of the Cys309 and thereby jeopardize oligomerization.

We therefore reinserted histidine at 310 to generate hIgG1-Fc-CL309/310CH-TP and investigated the consequence of this mutation on oligomerization and binding to human FcRn by ELISA (Figure 2). In contrast to the parent molecule, hIgG1-Fc-CL309/310CH-TP, bound human FcRn at pH 6.0 (Figure 2A), while having no detrimental impact on the ability of these molecules to oligomerize into hexamers (Figure 2B,C) or to interact with other effectors (see below).

### Hexa-Fc binds DC-SIGN in a valence dependent manner

**Figure 3:**
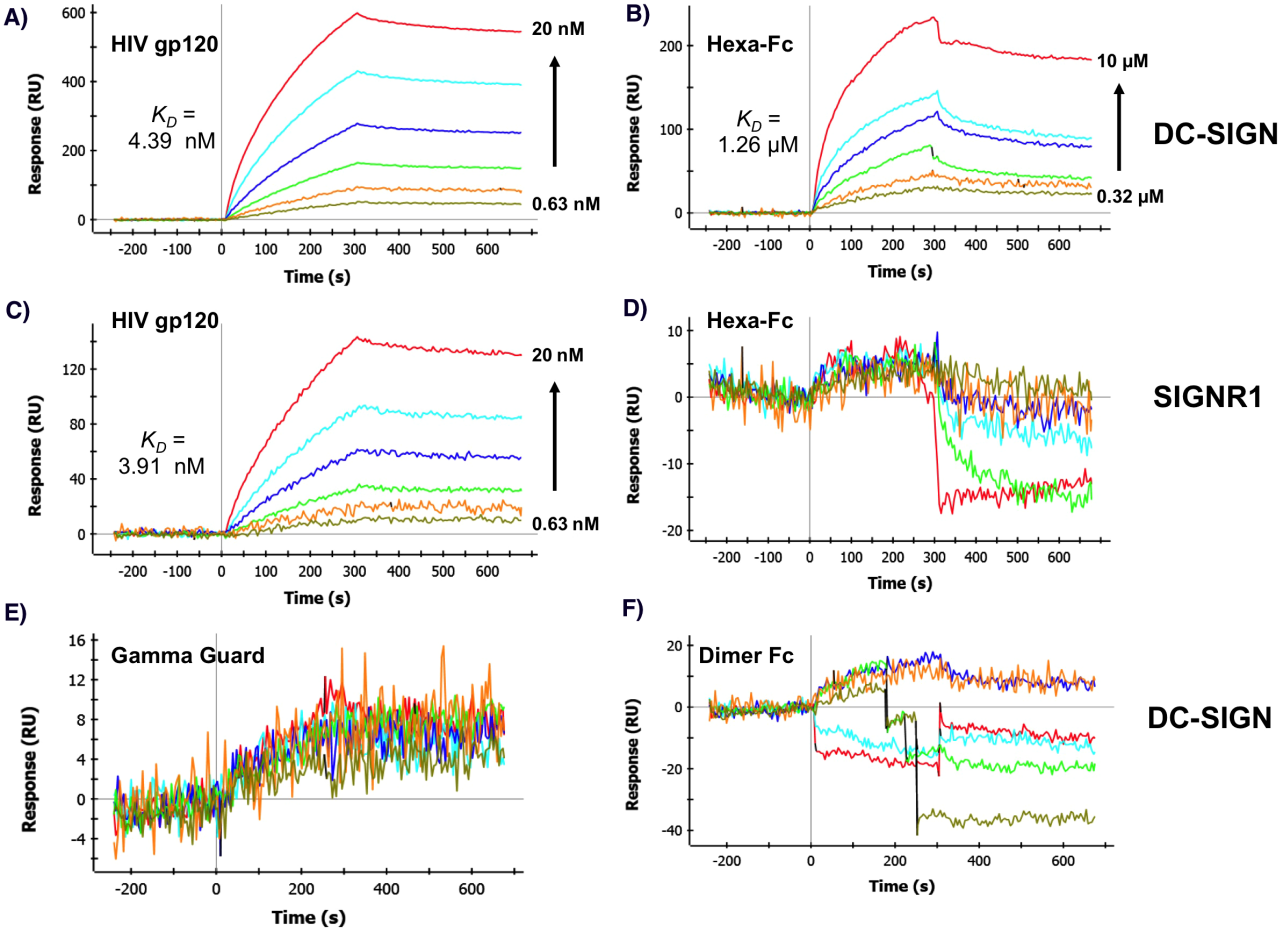
Binding of hexa-Fc to human DC-SIGN by multi-channel surface plasmon resonance analysis (MC-SPR). Association and dissociation curves of Igs binding to recombinant human DC-SIGN immobilized on a sensor chip. Hexa-Fc (panels b,d), IVIg GammaGard (panel e), dimeric-Fc (panel f) or gp120 control (panels a,c) were injected at doubling dilutions as indicated into flow at time 0, and replaced with buffer at 300 sec. Data are representative of duplicate experiments.

To test if hexa-Fc could bind to other, non-classical Fc-receptors that are also believed to be involved in controlling disease^9^, we investigated the interaction of hexa-Fc with soluble recombinant human DC-SIGN tetramers by multichannel surface plasmon resonance analysis (MC-SPR)^26^,^27^. Indeed, the sensorgrams show that hexa-Fc binds to DC-SIGN with moderate affinity (*K_D_* of 1.26 μM) in a dose-dependent fashion (Figure 3B). This interaction was stronger than that to dimeric-Fc, likely owing to the greater valency of the hexa-Fc. We also observed that the binding of hexa-Fc to DC-SIGN was stronger than that of IVIG GammaGard® (Figure 3E). Finally, we note that we did not detect any significant interactions between hexa-Fc and SIGNR1 (Figure 3D), the mouse orthologue of the human DC-SIGN, whereas the control HIV gp120, a well-studied DC-SIGN ligand known to carry substantial amounts of *N*-linked high mannose oligosaccharides, bound to both DC-SIGN and SIGNR1 (Figure 3AB), as previously reported^8^. The gp120 bound to DC-SIGN and SIGNR1 with high affinity and slow off-rates were observed consistent with the avidity associated with the clustering of carbohydrate-recognition domains within oligomers^26^. For the DC-SIGN-gp120 interaction, the *K_D_* was determined to be 4.39 nM (*k_on_* = 3.6 × 10^4^ M^−1^s^−1^; *k_off_* = 1.58 × 10^−4^ s^−1^). For SIGNR1-gp120 interactions, the *K_D_* was 3.91 nM (*k_on_* = 2.77 × 10^4^ M^−1^s^−1^; *k_off_* = 9.46 × 10^−5^ s^−1^). For hexa-Fc binding to human DC-SIGN, the overall affinity was lower when compared with gp120. This is to be expected from the lower density of favoured high-mannose glycans on the Fc polypeptide. However, the measured off-rate was still relatively slow, indicating that once bound, the hexa-Fc-DC-SIGN complex is stable. The *K_D_* was measured to be 1.26 μM (*k_on_* = 6.68 × 10^2^ M^−1^s^−1^; *k_off_* = 8.39 × 10^−4^ s^−1^). We note that the hIgG1-Fc-CL309/310CH-TP mutant also bound DC-SIGN and that a monomeric-Fc did not bind in these experiments (Fig. S4A).

### IgM-Fc and IVIG enriched for oligomeric Igs also bind DC-SIGN

As binding of hexa-Fc to DC-SIGN appeared to be partly owing to its high valency, we hypothesised that DC-SIGN binding may be a shared property of other oligomeric antibodies. To explore this possibility, we investigated the binding of a CHO cell derived recombinant (hexameric) IgM-Fc^18^ to DC-SIGN and SIGNR1 (Figure S5). The sensorgrams reveal that IgM-Fc binds to DC-SIGN with nanomolar affinity (*K_D_* of 0.26 μM). Further, in contrast to hexa-Fc, the IgM-Fc also bound strongly to SIGNR1 (*K_D_* of 2.2 μM). To test if this binding could be recapitulated with native antibodies, we investigated binding of Pentaglobin®, a clinically available IVIG preparation used in the treatment of sepsis and enriched for oligomeric Igs (12% IgM, 12% IgA and 76% IgG by weight). In contrast to IgM-Fc, Pentaglobin® bound human DC-SIGN but not SIGNR1 (Figure S5), a finding that may be attributed to differences in N-glycans or other undetermined posttranslational modifications that arise when expressing proteins in CHO cells.

### Hexa-Fc and IVIG exhibit differential glycosylation patterns

**Figure 4:**
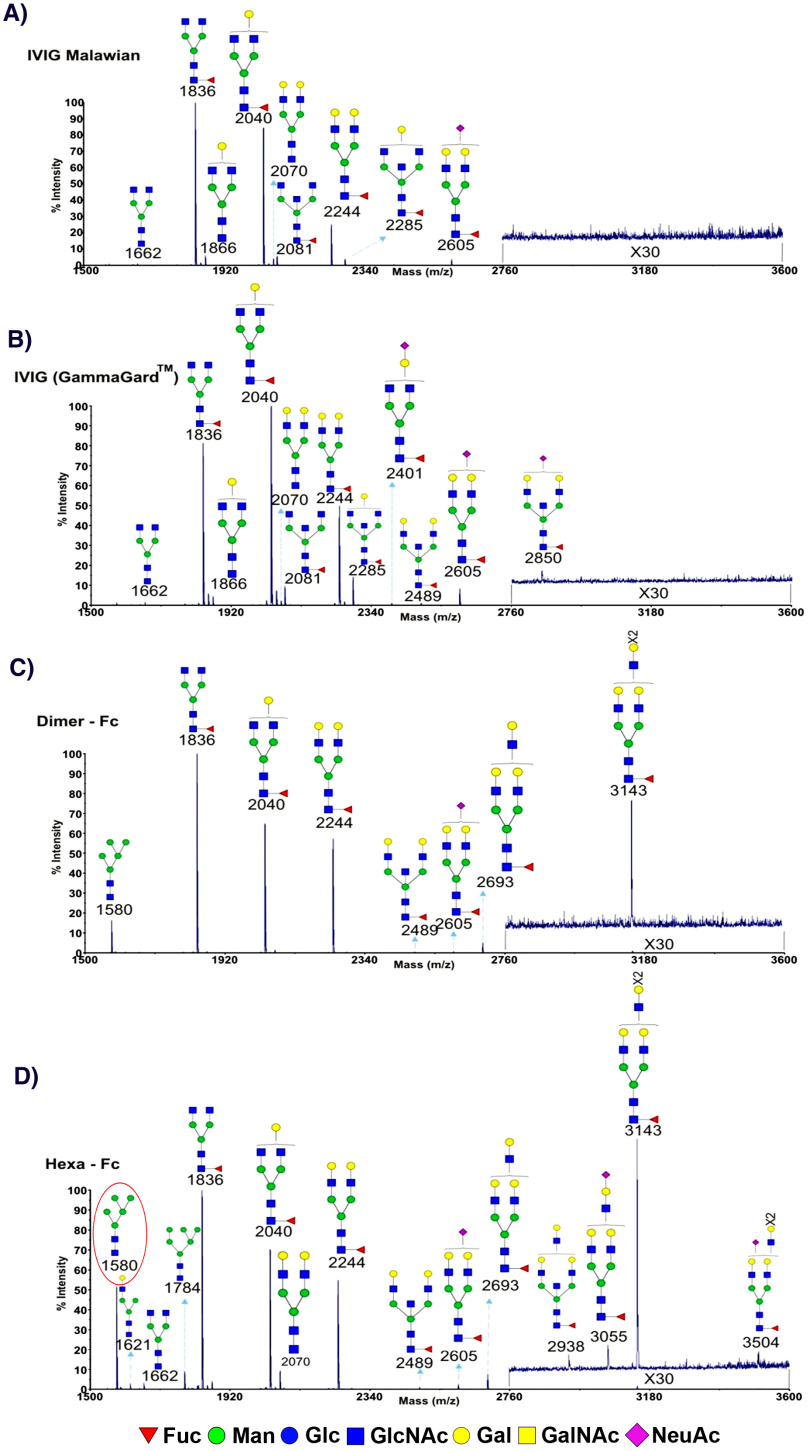
*N*-glycan profile of hexa-Fc and two different IVIg preparations. MALDI-TOF mass spectra of permethylated N-glycans of (a) IVIg from Malawians, (b) IVIg GammaGard, (c) dimeric Fc, and (d) hexa-Fc were obtained from the 50% MeCN fraction from a C18 Sep-Pak column (methods). Annotated structures are according to the Consortium for Functional Glycomics guidelines. All molecular ions are [M + Na]^+^. Putative structures are based on composition, tandem MS/MS, and biosynthetic knowledge. Due to the presence of heterogeneous multiantennary structures with extended LacNAc repeats, the annotations are simplified throughout by using biantennary structures with the extensions listed in parentheses. Structures that show sugars outside a bracket have not been unequivocally defined. Circled in red is the sugar modeled in the MD simulations.

Since the interaction of IVIG with FcRL5^22^, FcγRIIb^28^ and DC-SIGN^9^,^11^,^29^ has been attributed to direct and/or indirect effects of sialic acid on the Fc, we next investigated the nature of the *N*-glycans on hexa-Fc and compared them with two different IVIG preparations (GammaGard™ or Malawian IVIG) and the dimeric-Fc (Figure 4). MS analysis of hexa-Fc revealed a paucity of sialylated structures but enrichment for high mannose glycans (Man_5_GlcNAc_2_, Man_6_GlcNAc_2_) (Figure 4D). This glycan profile is also consistent with observations that DC-SIGN binds high mannose structures. MS/MS fragmentation was performed on ions whose masses were consistent with the presence of fucose in order to determine whether hexa-Fc contains terminal antennal fucose residues such as in the Lewis X antigen which can also bind DC-SIGN. These experiments ruled out antennal-linked fucose. For example, MS/MS of m/z 2244 shows a core rather than terminal location for the fucose (Figure S6), indicating that the DC-SIGN binding affinity for hexa-Fc is likely the result of increased avidity binding mediated by mannose. The MS analysis also revealed hexa-Fc to be richer in larger multi-antennary and polylac containing N-glycans (for example m/z 2693, 3143 and 3504) which would present more terminal galactose when compared to IVIG N-glycans (Figure 4D).

### DC-SIGN binding of hexa-Fc is critically dependent on the presence of N-linked glycans

To confirm that the interaction between DC-SIGN with hexa-Fc and IgM is dependent on the presence of N-glycans, these carbohydrates were removed from hexa-Fc, IVIG, and human IgM with peptide *N*-glycosidase (PNGase) F (Figure S7), and their resulting ability to bind human DC-SIGN investigated by enzyme-linked immunosorbent assay (ELISA) (Figure S8A). De-glycosylated hexa-Fc was indeed unable to bind DC-SIGN, demonstrating that binding by hexa-Fc was fully dependent on a PNGase F susceptible glycan(s). By contrast, there was ~50% and ~30% residual binding seen with PNGase F treated human IgM and IVIG, respectively (Figure S8A).

To provide further information about the identity of the glycans on hexa-Fc mediating this interaction with DC-SIGN, we first examined the effects of treating hexa-Fc with the endoglycosidase, Endo S, which earlier work showed removes complex-type N-linked glycans (as expected for those containing sialic acid) but not oligomannose glycans from native (not denatured) human IgG^30^. We found that Endo S treatment did not affect binding of hexa-Fc to DC-SIGN (Figure S8B), which suggests that the hexa-Fc/DC-SIGN interaction is mediated by oligomannose glycans. We also performed experiments using Endo H, which specifically cleaves oligomannose glycans but not complex glycans^30^, although this activity often requires denaturation of the glycoprotein to enable access of this enzyme to the attached glycans. Indeed previous work has shown that Endo H does not remove glycans from native human IgG, a finding that we also confirm here for hexa-Fc (Fig. S7)^30^. We found that Endo H treatment did not reduce binding of hexa-Fc to DC-SIGN (Figure S8B), which may reflect a lack of enzyme accessibility for the attached glycans, as observed here for hexa-Fc (Fig. S7) and previously for IgG^30^,^31^,^32^. Indeed, binding of hexa-Fc, and to a lesser extent IVIG to DC-SIGN increased in the presence of Endo H, a finding that may reflect non-enzymatic aggregation of IgG caused by Endo H in the current ELISA-based assay.

We also examined the effects of deleting the N297 glycosylation site by mutagenesis to alanine (N297A mutant, Figure S4D). This modification led to proteins unable to bind DC-SIGN (Figure S4A) or activate complement (Figure S4C). The N297A mutant is still capable of forming higher order oligomers (Figure S4E), as is known for aglycosylated polymeric IgG^33^, confirming that binding to DC-SIGN and C1q is critically dependent on the presence of the glycan and not the increased valence of the Fc per se.

### Hexa-Fc binds complement C1q and activates complement via the classical pathway

**Figure 5:**
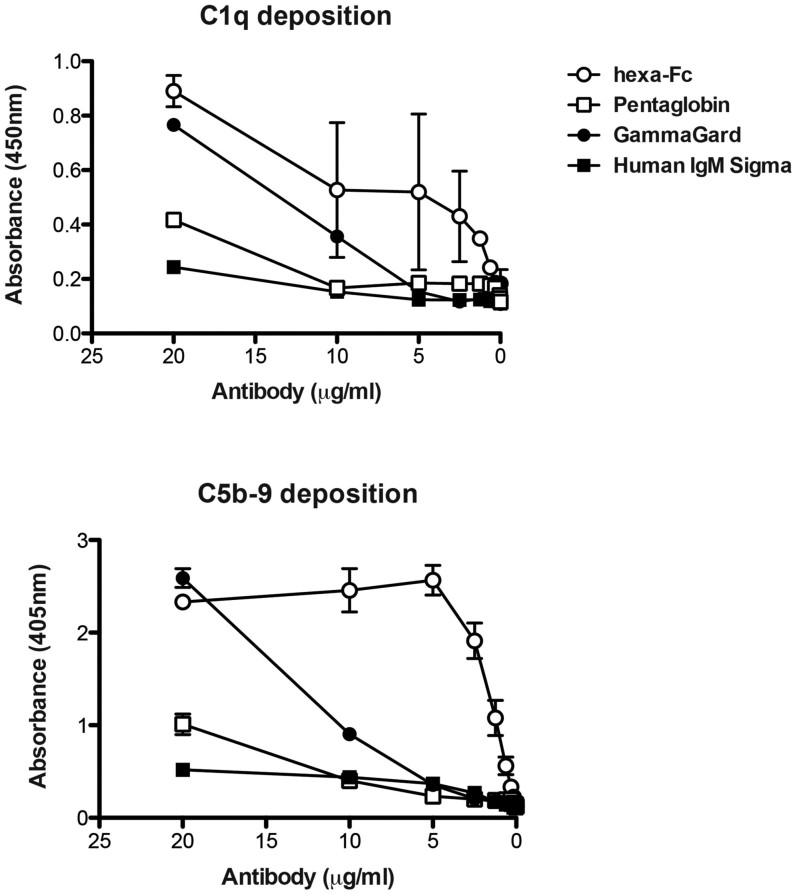
Hexa-Fc binds C1q and activates the classical pathway. (a) C1q and (b) C5b-9 deposition to antibodies as detected by ELISA. The figure shows the mean of three independent experiments (±SD).

A common mode of action of anti-tumour monoclonal antibodies is complement-dependent cytotoxicity (CDC), in which direct interaction of surface Ag-bound IgG with complement C1q triggers cell death through CDC^19^. Noncovalent interactions between Fc segments of IgG have recently been shown to result in the formation of ordered IgG hexamers after antigen binding on cells^19^,^34^. These IgG hexamers recruited and activated the complement cascade and could be further engineered into therapeutic IgGs for enhancement of complement activation and killing of target cells^19^. By nature of its oligomeric structure, we wondered if hexa-Fc may also activate complement and thereby open up the scaffold for oncology- or vaccine-directed approaches. Binding of C1q and activation of the classical complement pathway was assessed using ELISA. Hexa-Fc bound C1q more efficiently than either IVIG or IgM (Figure 5, upper panel), a finding that was also reflected in their ability to activate complement to its terminal C5b-9 components (Figure 5, lower panel). The hIgG1-Fc-CL309/310CH-TP mutant also bound C1q and enabled C5b-9 deposition as efficiently as the wild-type hexa-Fc molecule (Figure S4B). A monomeric-Fc control or an oligomeric control lacking N297 glycans did not bind C1q or permit C5b-9 deposition in these same experiments (Figure S4B,C).

### Modelling of hexa-Fc binding to inhibitory receptors, FcRL5 and DC-SIGN

The results described above indicated two observations that, based on previous work, were somewhat unexpected: namely, the binding of FcRL5 to hexa-Fc demonstrated that this interaction did not require the presence of Fab or F(ab′)_2_ domains^22^ and the binding of hexa-Fc to DC-SIGN appeared to be mediated by mannose-containing glycans^9^. We sought structural insight into these observations by extensive all-atom molecular dynamics (MD) simulations.

**Figure 6:**
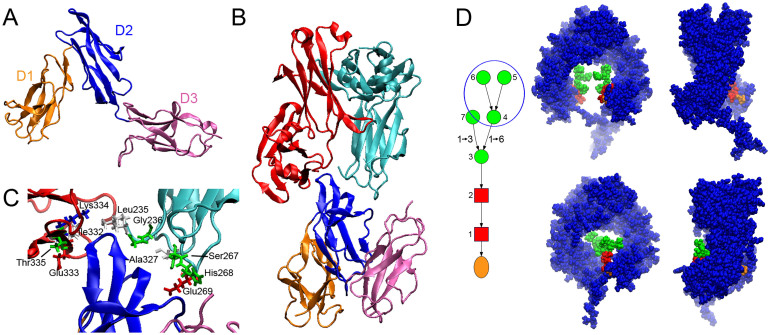
Structure of FcRL5, Fc/FcRL5, and glycosylated Fc domain determined from homology modeling and MD simulations. (a) Shown is the structure of the FcRL5 model near the end of equilibration simulations, showing well formed secondary and tertiary structures that are expected from the crystal structure of FcγRI, which was used as the initial template. (b) Overview of the structure of the Fc/FcRL5 complex, with the Fc colored red and cyan and the FcRL5 colored as in (a). The known structure of Fc/FcγRIII was used to initially position FcRL5 in contact with the Fc domain. (c) Detailed view of the contact region of the Fc/FcRL5 complex. The Fc residues that are frequently within 3Å of FcRL5 near the end of the equilibration simulation are shown to give a sense of the number and scope of contact region. Although these proteins remained in contact for the duration of the simulations, the contact was weaker than that in the Fc/FcγRIII complex (Figure S10). (d) The upper panel is the initial structure of the glycosylated Fc domain, where the atoms of the complex are depicted as van der Waals spheres. Shown in blue is the hFc, while the colors for the sugars are as depicted in the schematic of the Man_5_GlcNAc_2_ glycan shown on the left, where mannose residues are circles, the N-acetylglucosamines are squares, and the asparagine residue is an oval. Two views of the complex, differing by 90° rotation about the long axis, are shown. The lower panel shows the monomer after ~125 ns. In this, one glycan chain remains closely associated with the C_γ_2 domain and remains buried within the cavity. However, the other chain has adopted a structure that interacts with the C_γ_2 domain only via the di-N-acetylchitobiose core. In this more loosely bound configuration, the α1–6 mannose branch residues of the glycan (circled in the schematic) are near to the cavity entrance, and therefore more accessible to potential interactions with lectins such as DC-SIGN.

For the former, we first generated a model of human FcRL5 based on its high homology to FcγRI^35^, whose structure is known^36^. Following extended equilibration simulations (>40 ns), the model was found to adopt an architecture of stable secondary and tertiary structure consistent with expectations based on the FcγRI model (Figure 6A). One notable difference though is the relative disposition of the D1 and D2 domains, which exhibits a hinge angle of ~35° in the FcγRI crystal structure yet is ~50° in this FcRL5 structure (Figure S9). We verified with simulations of a similar duration that the D1–D2 hinge angle in FcγRI maintains a lower value (~30°) for the duration of the simulations (Figure S9). However as discussed previously^36^, the D1-D2 hinge angles of low affinity FcγRs, FcγRII and FcγRIII, are also much larger than FcγRI (52°–55°), and such a sharply bent D1/D2 structure might only be a feature of high affinity FcγRs. Hence, a larger D1/D2 hinge angle in the low affinity FcRL5 is consistent with other low affinity FcγRs, and its observation in the equilibrated FcRL5 model here thus provides further support for its accuracy.

We then placed this equilibrated FcRL5 structure in the analogous position of FcγRIII in the known structure of FcγRIII^37^ and performed extensive equilibration simulations (>120 ns). For comparison, we also performed similarly long simulations on the Fc/FcγRIII complex. Despite the lack of Fab domains, FcRL5 remained in contact with the Fc domain for the duration of the simulations (Figure 6B,C, see supplementary movie 1). As with FcγRIII^37^, FcRL5 interacted with both Fc heavy chains, one predominantly in the D1/D2 junction and the other within the D2 domain, although the number of these associations were significantly lower than in the FcγRIII/Fc complex (Figure S10). In particular, the heavy chain interaction with the D1/D2 junction in FcRL5 was markedly weaker than in FcγRIII complex (see supplementary movie 2). Hence, these findings suggest that indeed FcRL5 can interact with just the Fc domain but this interaction is weaker than that of FcγRIII/Fc.

As for the putative interactions between mannose-glycans and DC-SIGN, as a first step towards a structural understanding of this association, we noted that there were crystallographic data of a human Fc domain with high mannose glycans^38^. Using this structure as a template, we constructed a model of the human Fc domain (mutated in two residues to enable oligomerization into the hexa-Fc) containing the Man_5_GlcNAc_2_ glycan that MS identified here (Figure 4) as attached to hexa-Fc^18^, and evaluated the structure with equilibration MD simulations.

Immediately apparent with this initial structure however was the limited accessibility of the mannose residues for any putative lectin: the entrance to the internal cavity of the Fc domain (where the glycans are located) is roughly elliptical, with dimensions of 2 nm × 3.5 nm and the mannose residues are deeply buried within this cavity (Figure 6D, upper panel). With each carbohydrate recognition domain (CRD) of the tetrameric DC-SIGN shaped as a sphere of 3 nm diameter^39^, this Fc-glycan structure poses significant limitations for potential interactions with DC-SIGN.

However, once equilibrated, the complex frequently adopted a configuration in which the α1–6 branch mannose residues that are expected to interact with DC-SIGN^39^ are located near the entrance to the Fc cavity (Figure 6D, lower panel, see supplementary movies 3 and 4). During the simulation, both N-glycan chains essentially adopt one of two configurations: one in which the di-N-acetylchitobiose core, the central β mannose, and α1–6 branch residues are all in close proximity to the C_γ_2 domain (similar to the glycan structures observed in earlier crystallographic studies^38^,^40^,^41^) and a previously uncharacterized configuration in which only the di-N-acetylchitobiose core is close to the C_γ_2 domain. The α1–3 branch mannose residue in both configurations is essentially always oriented towards and frequently interacting with the other glycan chain. While one glycan chain predominantly adopted only the former structure (96.7% of the trajectory, see Methods), the other chain frequently adopted the latter configuration (34.5% of the trajectory), and it is in this latter configuration that the α1–6 branch mannose residues were localized near to the entrance of the cavity (Figure S11). We verified that at this location these mannose residues are indeed accessible to potential interactions with DC-SIGN (Figure S12). We note that this Fc structure can also be assembled into a barrel-shaped hexameric architecture of the hexa-Fc following the structural principles previously described^18^ (Figure S1).

## Discussion

Previous studies suggested that FcRL5 might be a receptor for IgG^42^,^43^. However, binding of soluble monomeric IgG to FcRL5-transfected 293 cells was not observed in FACS-based assays, indicating that FcRL5 was likely to be a low- to medium-affinity FcR, if at all^6^. Our data clearly show that FcRL5 can bind complexed IgG1 and that this interaction can occur in the absence of the Fab or F(ab′)_2_ regions, as hexa-Fc does not contain these domains. MD simulations further show that this interaction can involve similar Fc regions as implicated in the interaction with classical FcγRs^22^,^37^,^44^, although the number and strength of these associations is noticeably lower than at least the FcγRIII/Fc complex. This suggests that the observed significant binding of FcRL5 to hexa-Fc is likely owing to the oligomeric nature of the complex, which would explain the failure to observe the aforementioned FACS-based assays with monomeric IgG^6^.

These findings are in agreement with two recent publications that have studied the interaction of IgG with FcRL5^6^,^22^. One of these studies^22^ showed that a stronger interaction with monomeric IgG may involve contributions from both the IgG Fc and IgG F(ab′)_2_, unlike classical FcγRs such as FcγRIIb that only bind via the Fc^44^. Our observation of a stronger interaction of hexa-Fc to FcγRIIb than FcRL5 may thus be owing to an inherently stronger interaction with the Fc domain in FcγRIIb compared to FcRL5. The finding that heat-aggregated IgG binds more strongly to FcRL5 than to FcγRIIb, also consistent with previous work^6^,^22^, may thus be owing to the additional contact with F(ab′)_2_ that do not occur in the FcγRIIb interaction.

DC-SIGN signalling invokes IL-10 production which is of significance in anti-inflammatory pathways^45^,^46^. Recent work showing that DC-SIGN and SIGNR1 are important receptors in the efficacy of IVIG in controlling autoimmune disease^9^,^11^ prompted us to investigate the interaction of these additional receptors with hexa-Fc (Figure 3). Indeed, we found that hexa-Fc bound more strongly to DC-SIGN than monomeric IgG, and that this interaction was wholly dependent on N-glycans as their removal with PNGase F, or via mutagenesis of N297 to alanine, resulted in molecules unable to bind the receptor (Figures S4, S7 and S8). The interaction of IgG with DC-SIGN has recently been ascribed to terminal sialylation of the N-linked glycan at position 297 in the Fc^9^. Contrary to expectations though, we found that the glycans attached to hexa-Fc were generally more diverse, being rich in both terminal mannose and galactose, and that terminal sialylation was rarely observed (Figure 4). Further, the results with Endo S and H, although not completely definitive (owing to the common requirement for glycoprotein denaturation of Endo H), are consistent with the involvement of high mannose glycans in this interaction.

Our tentative conclusion that mannose but not sialic acid is critical to binding of DC-SIGN by hexa-Fc is supported by the fact that α2,3 disialyl linkages applied by CHO (as with hexa-Fc) are apparently not involved in amelioration of autoimmune disease by recombinant Fc^11^,^13^,^29^. We also note that the role of sialic acid (but not mannose) in ITP^47^ and binding by DC-SIGN or SIGNR1 has been questioned by numerous recent studies^10^,^12^,^47^,^48^. Finally, our MD results provide a structural means by which an interaction mediated via mannose residues could occur, which appeared challenging based on available crystallographic data of glycosylated-Fc^40^.

Hexa-Fc also binds C1q and leads to C5b-9 deposition when coated down onto ELISA plates (Figure 5). This property may be useful for vaccines as complement activation is crucial for antigen retention on DCs and for the generation of long-lived memory B and T cell responses^49^,^50^. Complement activation may also be very useful in oncology settings if hexa-Fc can be specifically targeted to tumour cells, as recently demonstrated with conventional mAbs^19^. Currently, the presence of a fusion protein greatly interferes with the ability of hexa-Fc to engage complement and FcγRs^18^. The lack of binding to FcγRs and C1q is due to the fusion protein blocking access to the FcγR and C1q binding sites (the lower hinge region and the amino-terminal region of Cγ2 domains) or to a lack of receptor flexibility when fused in the existing hinge architecture^37^,^51^,^52^. It has long been considered that the hinge region serves as a spacer and mediates segmental flexibility allowing the fusion protein to assume a variety of orientations in space relative to the Fc^52^. Modifications to the existing hinge e.g. use of the extended hinge from human IgG3, may therefore move the fusion protein away from the critical FcγRs and C1q binding sites and thereby reinstate effector functions to hexameric Fc-fusions that are critical for tumour cell killing and clearance. However, where complement activation is neither desirable nor safe, e.g. when hexa-Fc is used as a biomimetic replacement for IVIG therapy in autoimmune disease, mutations that disrupt C1q binding e.g. K322A, P329A, P331A may be introduced into the wild-type molecule^53^.

A critical feature to the utility of oligomeric Fc-fusion proteins in future drugs or vaccines will be their ability to interact or not with FcRn. Here we have shown that His310 within the C_γ_2 domains is critical to binding of hexa-Fc to human FcRn (Figure 2), although whether this reinstates binding in the context of N-terminal fusions remains to be tested. Additional work now needs to be undertaken to determine if oligomeric Fcs and/or oligomeric-Fc-fusions will be internalized, recycled, transcytosed or degraded. Previous work has shown that FcRn is capable of transcytosing IgG immune-complexes efficiently across epithelial cells for enhanced degradation and presentation^23^,^54^,^55^. Previous work using oligomeric IgG1μtp or IgG4μtp with molecular weights >750 kDa has shown β-phase *in vivo* half-lives comparable to monomeric IgG3^56^. Indeed, clearance of these oligomeric IgGs resembles the clearance of IgM, with α-phase half-lives two to four times longer than that of IgM. Given the smaller size of hexa-Fc (~324 kDa), and with physical dimensions approximating monomeric IgG (Figure S1C) it may reasonably be anticipated that the *in-vivo* half-life of hexa-Fc may be greater than that of IgM. Where hexa-Fc is routed after FcRn binding remains to be determined and is currently under investigation.

The nature of the fused protein may also potentially affect FcRn binding and interactions with FcRn will therefore most likely need to be determined for each unique fusion. The introduction into hexa-Fc of additional mutations known to enhance or reduce interactions of the Fc with FcRn may further enhance its translational potential^57^,^58^. Hexa-Fc now provides a template molecule to further engineer selective gain-of and/or loss-of function mutations, as demonstrated here for FcRn, that allow the exisiting multimeric nanoscopic scaffold to be tailored for optimal use in novel drugs and vaccines.

## Methods

### Production of the CL309/310CH mutant

The generation of hexa-Fc has been previously described^18^. The CL309/310CH mutant was constructed by PCR overlap extension mutagenesis from the wild-type vector (pFUSE-hIgG1-Fc-TP-LH309/310CL) as the template using the internal mismatched primer mut-3:5′-ACCGTCTGCCACCAGGACTGG-3′ and its complement to incorporate a CTC to CAC substitution and Fcmut-1:5′-ACCCTGCTTGCTCAACTCT-3′ and Fcmut-1:3′-TTGATGAGTTTGGACAAACCA-5′ as flanking primers. The N297A mutant was similarly constructed from the same vector using the internal mismatched primer mut-3:5′-CTCGTCATGCGGTCGTGCATG -3′ and its complement to incorporate an AAC to GCC substitution and Fcmut-1:5′-ACCCTGCTTGCTCAACTCT-3′ and Fcmut-1:3′-TTGATGAGTTTGGACAAACCA-5′ as flanking primers. PCR products were then digested using *Bgl*II and *Nhe*I (New England Biolabs) and cloned back into the wild-type vector to generate pFUSE-hIgG1-Fc-TP-CL309/310CH or N297A. To verify incorporation of the desired mutation and to check for PCR-induced errors, the entire coding sequence of the new expression plasmid was sequenced on both strands. CHO-K1 cells (European Collection of Cell Cultures) were transfected with plasmid using FuGene (Promega) and positive clones selected, expanded and purified as previously described for hexa-Fc^18^. Monomeric-Fc, dimeric-Fc, and IgM-Fc were generated as described previously and IgG1 Eu numbering is used throughout^18^.

### Complement binding assays

Antibodies were coated down to ELISA plates (Nunc) in carbonate buffer pH9 (Sigma-Aldrich) at the indicated concentrations overnight at 4°C. Plates were then washed five times in PBS/0.1% Tween-20 (PBST) before adding normal human serum (NHS) at 1:100 in Veronal buffered saline containing 0.5 mM MgCl_2_, 2 mM CaCl_2_, 0.05% Tween-20, 0.1% gelatin and 0.5% BSA and incubated for 2 h at room temperature as described previously^18^. After washing as above, plates were incubated with a 1:500 dilution of mouse anti-human C5b-9 (Serotec) or peroxidase labeled sheep anti-human C1q (Serotec) in PBST/0.1% BSA for 1 h at room temperature. For C5b-9 plates were additionally incubated in anti-mouse IgG (Pierce) diluted 1:500 in PBST/0.1% BSA for 1 h prior to washing and developing with p-nitrophenyl phosphate substrate (Sigma). C1q ELISAs were developed with 3,3′,5,5′-tetramethylbenzidine dihydrochloride (Sigma) in phosphate citrate buffer containing sodium perborate (Sigma). After 10 min, colour development was stopped with 50 μl of 2 M H_2_SO_4_ and the absorbance at 450 nm read using an ELISA plate reader.

### Human FcRn binding assays

Microtiter wells (Nunc) were coated with titrated amounts of the Fc (20.0- 0.1 μg/ml) in PBS and incubated over night at 4°C prior to blocking with 4% skimmed milk (Acumedia) for 1 h at room temperature. The wells were washed four times with PBS/0.005% Tween 20 (PBS/T) pH6.0 before addition of GST-tagged human FcRn in 4% skimmed milk PBS/T pH6.0 and added to the wells^18^. After incubation for 2 h follwed by a washing as above, an horseradich peroxidase conjugated polyclonal anti-GST from goat (GE Healthcare) was added and incubated for 1 h. Wells were washed as above before 100 μl of 3,3′,5,5′-tetramethylbenzidine substrate (Calbiochem) was added to each well and incubated for 45 min before 100 μl of 0.25 M HCl was added. The absorbance was measured at 450 nm using a Sunrise TECAN spectrophotometer (TECAN, Maennedorf, Switzerland).

### N-glycomic analysis

N-glycomic analysis was performed according to a protocol described previously^59^. Briefly, 50 μg of each sample was reduced by dithiothreitol (Sigma, Aldrich) and then carboxymethylated by iodoacetic acid (Sigma Aldrich). Samples were subsequently dialyzed, freeze-dried and digested by trypsin (Sigma Aldrich). The peptides/glycopeptides were purified using Oasis HLB Plus Short cartridges (Waters). N-glycans were released from glycopeptides by PNGase F (Roche Applied Science) and isolated from peptides using Sep-Pak C18 catridges (Waters). The released N-glycans were permethylated, purified by Sep-Pak C18 cartridges again, freeze-dried and dissolved in 10 μl 1,5-dihydroxybenzoic acid in 70% (v/v) aqueous methanol; for MS/MS, 20 mg/ml 3,4-diaminobenzophenone in 75% (v/v) aqueous acetonitrile). MALDI-TOF MS analysis using a Voyager-DETM STR mass spectrometer (Applied Biosystems). The data were analyzed using Data Explorer (Applied Biosystems) and Glycoworkbench^60^.

### Immune cell binding assays

Peripheral blood mononuclear cells (PBMC) were purified from buffy coats kindly provided by human volunteers using Lymphoprep™ (Axis-Shield) according to manufacturers instructions. All work was conducted after approval by the ethical review committee of the Liverpool School of Tropical Medicine. One hundred thousand PBMCs were incubated with 200 μl FACs buffer (phosphate-buffered saline, 0.2% bovine serum albumin, 5% goat serum) containing 50 μg of hexa-Fc or buffer only as indicated for 1 h at room temperature. Cells were washed twice with FACs buffer and incubated for 1 h at 4°C with 1/500 dilution of F(ab′)_2 _goat anti-hIgG-Fc-phycoerythrin (PE), goat anti-hCD19-fluorescein isothiocyanate (FITC)-conjugated (BioLegend) and goat anti-hCD14-APC-Cy7 (BioLegend) in 200 μl FACs buffer. After washing with FACs buffer, cells were analyzed on a FACScan (BD Biosciences). Data acquisition was conducted with CELLQuest software (BD Biosciences) and the analysis performed with FlowJo version 9.1.

### FcRL5 and FcγRIIb binding assays

To test for hexa-Fc binding by FcRL family members, cDNA encoding human CD200R, FcRL4, or FcRL5 were ligated into pFLAG-CMV-3 (Sigma-Aldrich). cDNA encoding human FcγRIIb was ligated into pEF6 (Invitrogen)^6^. Proteins were expressed in 293 cells by transient transfection using Lipofectamine 2000^6^. Transiently transfected 293 cells were used for Ig binding assays 36–42 h after transfection. Purified human IgG1 was obtained from Sigma-Aldrich and hexa-Fc was purified as previously described^18^. For the heat aggregation assay Igs were aggregated by heating to 60°C for 30 min. Igs were then diluted to 100 μg/ml in PBS/1% BSA. The 293 cells were incubated for 30 min on ice with the Igs and washed four times, followed by incubation with biotin-conjugated goat F(ab′)_2 _anti-human IgG (Southern Biotechnology) for 20 min on ice. Cells were washed three times and incubated with FITC-conjugated anti-Flag Ab (M2; Sigma-Aldich) or isotype control as used previously for 20 min on ice^6^. To detect FcγRIIb-expressing cells, a PE-conjugated anti-human FcγRIIb Ab (Beckman Coulter) was added to FcγRIIb-transfected samples. Cells were washed twice and analyzed by flow cytometry on a FACSCalibur (BD Biosciences) for Ab binding. Dead cells were excluded by propidium iodide staining^6^,^61^.

### Multichannel surface plasmon resonance analysis

Recombinant human DC-SIGN tetramers were generated as described previously^26^. Recombinant SIGNR1 was from R&D systems. Purified recombinant HIV gp120 was a kind gift of Dr Max Crispin (University of Oxford). Soluble recombinant DC-SIGN and SIGNR1 proteins were captured on GLM sensor chips (Bio-Rad laboratories) via amine coupling with sulfo-N-hydroxysuccinimide/1-Ethyl-3-[3 dimethylaminopropyl]carbodiimide and all sensorgrams using soluble-phase analytes of Ig preparations were recorded at 25°C with the ProteOn XPR36 surface plasmon resonance biosensor (Bio-Rad laboratories) at a flow rate of 25 μl per minute. Kinetic parameters for protein-protein interactions were determined using the 1:1 Langmuir modeling algorithms included in the ProteOn Manager software suite (Bio-Rad Laboratories). GammaGard™ and Pentaglobin™ were kindly provided by Baxter Healthcare and Biotest UK respectively. The generation of IgM-Fc has been described previously^18^. Human serum IgM and IgG (Sigma Aldrich).

### Modelling hexa-Fc interactions with FcRL5 and DC-SIGN

The homology model of FcRL5 was constructed with the automated homology modeling tools in DeepView^62^, using the human FcRL5 (PDB accession no. Q96RD9) and the crystal structure of the FcγRI (PDB accession codes: 3RJD). The structure (and all models here) was then solvated in TIP3 water^63^ and then minimized and equilibrated using VMD/NAMD^64^ and the CHARMM36 force field^65^, in the constant pressure and constant temperature (NPT, 295K, 1atm) ensemble. The temperature and pressure were controlled by the Berendsen thermostat and barostate with a coupling time of 0.1 ps and 1.0 ps, respectively. The particle mesh Ewald algorithm was employed to treat electrostatic interactions. The van der Waals interactions were treated with a cut-off of 12Å, and the integration step was set to 2 fs. After ~10 ns, the protein attained an equilibrated conformation, as judged by the root-mean-square deviation of the protein backbone. The protein secondary and tertiary structures were evaluated with VMD. A similar procedure was followed for the simulations of FcγRI. The D1–D2 hinge angle was determined by measuring the angle subtended by residues Val81, Ala88, and Ala94 in FcRL5 and Ile96, Gly103, and Ser110 in FcγRI. For the model with Fc, the crystal structure of the FcγRIII/Fc (PDB accession codes: 1E4K) was used as a template. The equilibrated structure of FcRL5 was superimposed on the FcγRIII structure, aligning the D1 and D2 domains of FcRL5 with the corresponding domains in FcγRIII, using the least-squares fitting procedure in DeepView. The Fc domain used in these simulations was the human Fc structure of the FcγRIII/Fc complex.

For the simulations of the mannosylated IgG, the crystal structure of the human IgG1 (PDB accession codes: 2WAH) was used as the template and initial structure for the model, as it contained high mannose glycans. However the glycans present in this structure (namely, Man_9_GlcNAc_2_) are not attached to hexa-Fc (Figure 4). Instead, we studied the Man_5_GlcNAc_2_ glycan that is attached to hexa-Fc (circled in Figure 4D). To generate the initial structure of the Man_5_GlcNAc_2 _glycans for these simulations, we manually removed the appropriate mannose residues from the Man_9_GlcNAc_2_ structure. This new glycan structure was then attached to both heavy chains of the Fc domain. Finally, the hinge and two residue mutations that enable oligomerization into the hexa-Fc^18^ were generated. The initial files for the simulation were obtained with Glycan Reader^66^. The simulations ran for ~150 ns, and the trajectory analyzed after the protein had equilibrated after the first 30 ns. To distinguish between the two configurations described in the text, the number of C_γ_2 domain residues with 3Å (roughly, hydrogen-bonding distance) of glycan residues 2 through 6 (see Figure 6D) was calculated throughout the trajectory. Those structures with 2 or fewer residues within this distance were considered as the configuration more loosely associated with the C_γ_2 domain.

## Supplementary Material

Supplementary Information Supplementary Movie 1

Supplementary Information Supplementary Movie 2

Supplementary Information Supplementary Movie 3

Supplementary Information Supplementary Movie 4

Supplementary Information Supplementary Information
