# Supplementary material for: Developing the IVIG biomimetic, Hexa-Fc, for drug and vaccine applications
Source: Sci Rep. 2015 Apr 27;5:9526. doi: 10.1038/srep09526 (PMC5224519; doi:10.1038/srep09526)
Supplement: Supplementary Information [file srep09526-s5.pdf]

Supplementary information for

Developing the IVIG biomimetic, Hexa-Fc, for drug and vaccine applications

Daniel M. Czajkowsky[1], Jan Terje Andersen[2], Anja Fuchs[3], Timothy J. Wilson[3], David Mekhaiel[4], Marco Colonna[3], Jianfeng He[1], Zhifeng Shao[1], Daniel A. Mitchell[5], Gang Wu[6], Anne Dell[6], Stuart Haslam[6], Katy A. Lloyd[4], Shona C. Moore[4], Inger Sandlie[2,7], Patricia A. Blundell[4] and Richard J. Pleass[4*]

1 – Bio-ID Center, School of Biomedical Engineering, Shanghai Jiao Tong University, Shanghai, 200240 P. R. China.

2 - Centre for Immune Regulation (CIR) and Department of Immunology, Oslo University Hospital Rikshospitalet, P.O. Box 4956, Oslo N-0424, Norway.

3 - Department of Pathology and Immunology, Washington University School of Medicine, St. Louis, MO 63110, USA

4 - Liverpool School of Tropical Medicine, Pembroke Place, Liverpool, L3 5QA, UK

5 - Clinical Sciences Research Laboratories, Warwick Medical School, University of Warwick, Coventry CV2 2DX, UK

6 - Department of Life Sciences, Imperial College London, South Kensington Campus, London SW7

7 CIR and Department of Biosciences, University of Oslo, N-0316 Oslo, Norway

*Corresponding author

E-mail address: rpleass@liv.ac.uk

Telephone: +44 151 705 3315

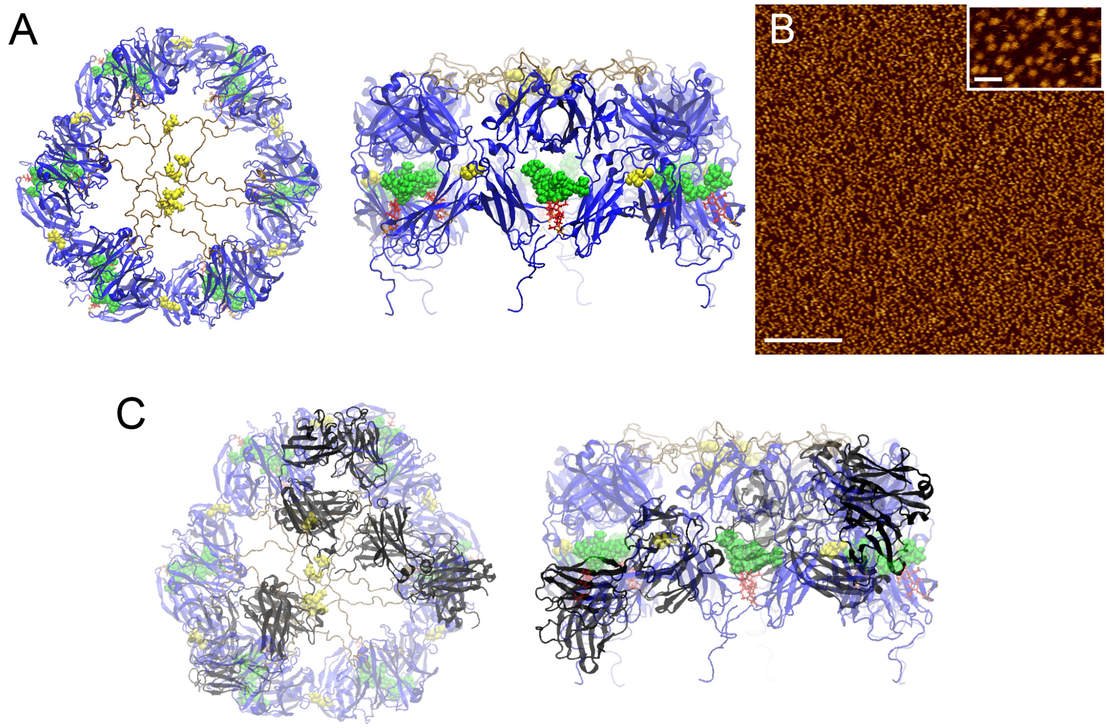

Figure S1. Structural characterization of Hexa-Fc. (**A**) Shown are top and side views of the hexamer with the C309 and tailpiece cysteine (here, C366) residues as yellow van der Waals (vdW) spheres. The C309 residues in neighboring monomers are covalently linked to each other in this model. The tailpiece cysteines in randomly selected monomers are also joined together. The tailpieces are shown in brown, the Fc of hIgG1 in blue, the sugars that are not mannose in red and the mannose residues as green spheres. (**B**) Tapping mode atomic force microscopy (AFM) images of hexa-Fc show birdcage structures of a size and shape consistent with the modeled hexameric complexes. Scale bar: 500nm, main figure; 60nm inset. **(C)** Superimposition of IgG1 b12 (pdb 1HZH) (grey) onto hexa-Fc (faded blue).

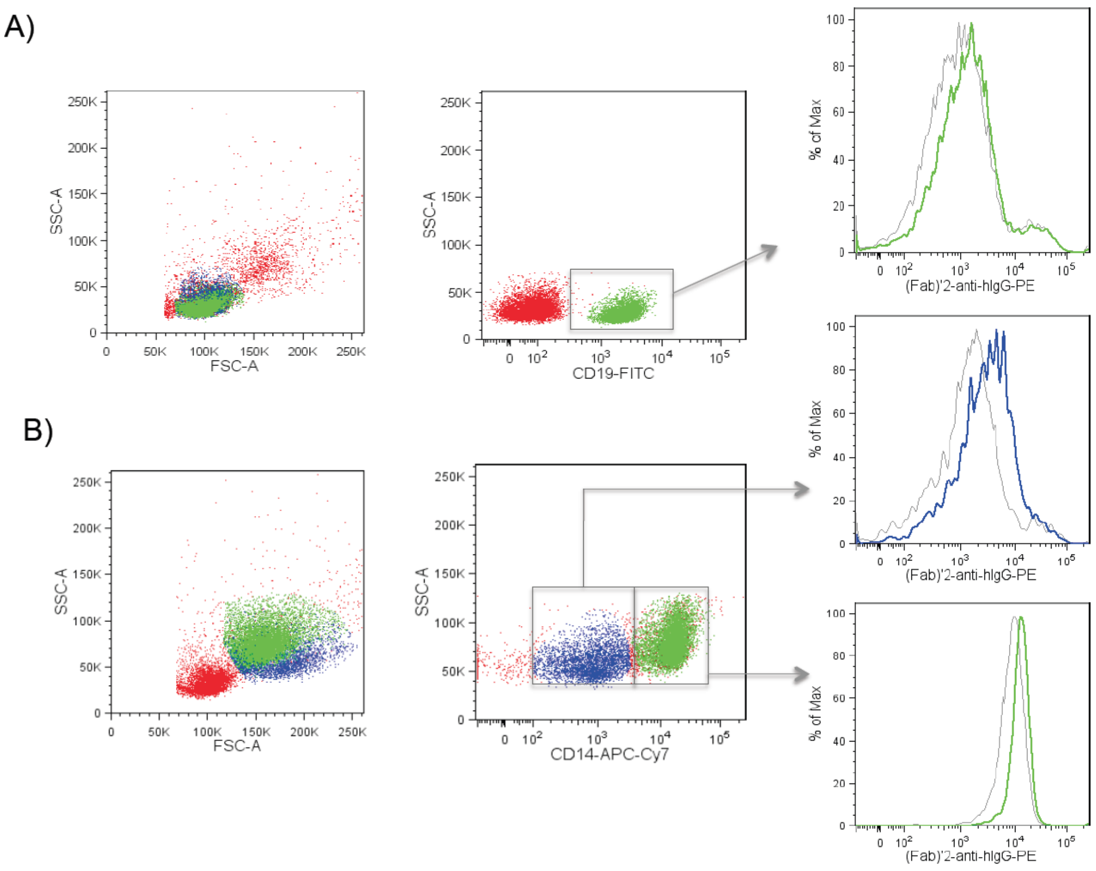

Figure S2. Hexa-Fc binds to CD19$^+$ human B cells and CD14$^+$ monocytes. Characteristic FACs plot showing different populations of human PBMCs represented by forward (*FSC*) and side (*SSC*) scatter profiles (left panel). **(A)** Individual CD19$^+$ B cells stained with anti-human CD19-FITC (shown by green dots) were gated (middle panel). Binding of 50μg of hexa-Fc (green trace) to gated human CD19$^+$ B lymphocytes was detected using phycoerythrin (PE)-labeled goat (Fab'$_2$) anti-human IgG (right panel). **(B)** Individual CD14$^+$ monocytes stained with anti-human CD14-APC-Cy7 (green dots for high CD14 expression and blue dots for low CD14 expression) were gated (middle panel). Binding of 50 μg of hexa-Fc (green trace for CD14$^+$ high, blue trace for CD14$^+$ low) to gated human CD14+ monocytes was detected using phycoerythrin (PE)-labeled goat (Fab'$_2$) anti-human IgG (right panels). Background binding detected with the PE-labeled goat (Fab'$_2$) anti-human IgG in the absence of hexa-Fc (grey traces). Data are representative of three separate independent experiments.

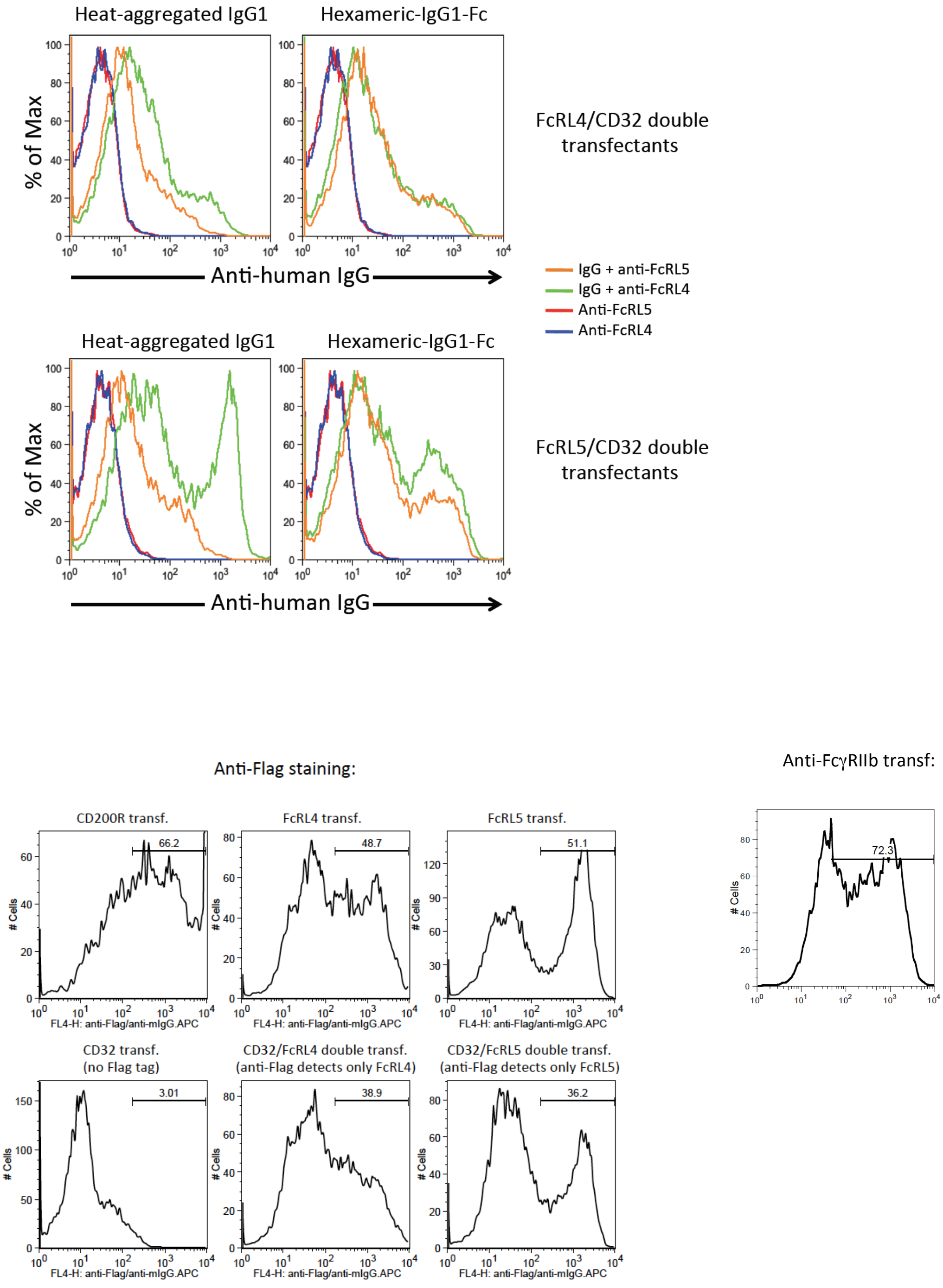

Figure S3. Hexa-Fc preferentially binds FcγRIIb over FcRL5. FcRL4/CD32 (top panel) or FcRL5/CD32 (bottom panel) double-transfectants were pre-incubated with anti-FcRL5 blocking mAb 509F6 (orange trace) or anti-FcRL4 blocking mAb 413D12 (green trace). Anti-FcRL5 blocking antibody did not reduce binding of hexa-Fc but markedly reduced binding of heat-aggregated IgG, showing that hexa-Fc prefers to bind FcγRIIb when given the choice of either receptor. Red and blue traces show binding by 509F6 and 413D12 respectively in the absence of human Igs. Cell surface expression of FcRL5 and FcRL4 were confirmed using FITC-conjugated anti-FLAG M2 mAb. The FcγRIIb construct was not FLAG tagged. We therefore confirmed they were FcγRIIb positive with an anti-FcγRIIb antibody and/or isotype matched controls (methods). Data are representative of duplicate experiments.

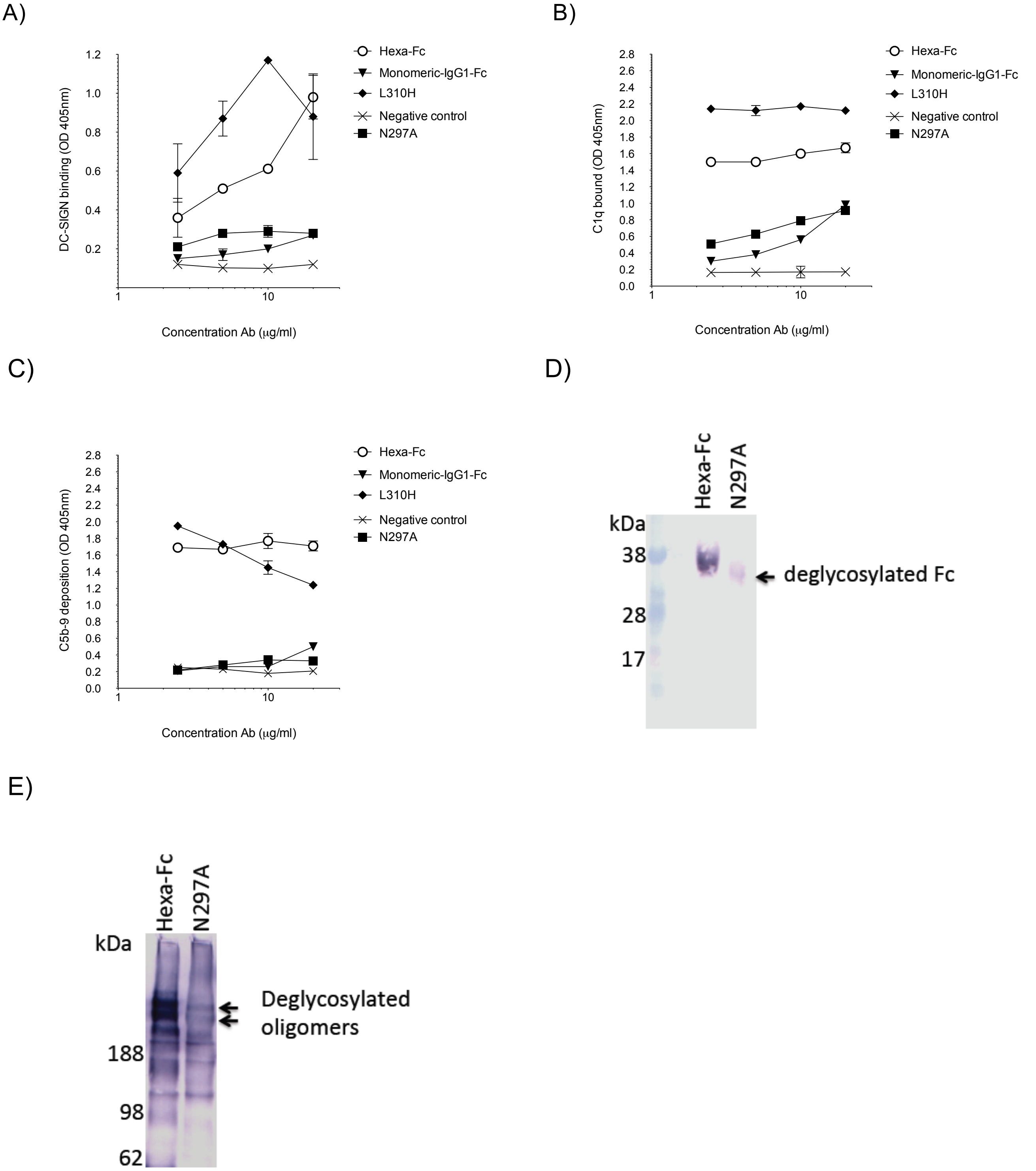

Figure S4. The hIgG1-Fc-CL309/310CH-TP binds DC-SIGN, C1q and activates the classical pathway. (A) ELISA data showing binding of the CL309/CH310CH to DC-SIGN. (B) C1q and (C) C5b-9 deposition to antibodies as detected by ELISA. An additional mutant hIgG1-Fc-N297A-TP in which the N-linked attachment site was removed by mutagenesis is no longer capable of binding DC-SIGN, C1q or of activating the classical pathway. (D) Loss of the glycan from N297A was confirmed by SDS-PAGE after reduction. (E) The hIgG1-Fc-N297A-TP mutant was still capable of forming higher order oligomers as determined by SDS-PAGE analysis under non-reducing conditions. These were of lower molecular weight when compared to hexa-Fc as a consequence of the loss of multiple sugars. The graphs show mean (±SD) of two independent experiments. The DC-SIGN binding assay is as described in Fig. S8.

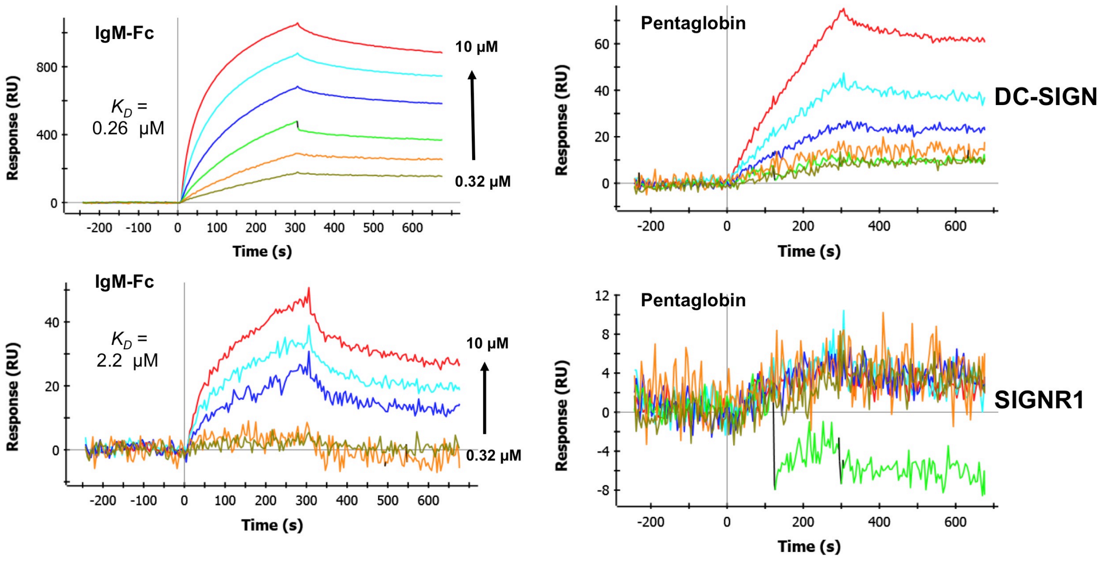

Figure S5. Binding of IgM-Fc to human DC-SIGN by multi-channel surface plasmon resonance (MC-SPR). Association and dissociation curves of IgM-Fc and Pentaglobin™ binding to recombinant human DC-SIGN or SIGNR1 immobilized on a sensor chip. IgM-Fc or Pentaglobin™ were injected at doubling dilutions from 10μM to 0.32μM into flow at time 0, and replaced with buffer at 300sec. Data are representative of duplicate experiments.

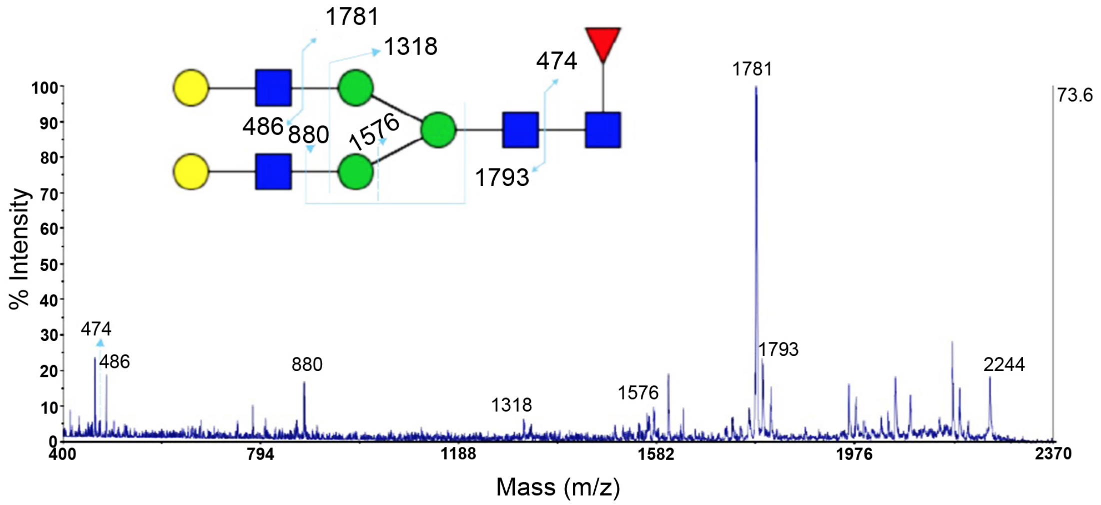

Figure S6. MALDI-TOF-TOF fragment ion spectra obtained after collisional activation of the molecular ion at m/z 2244 showed a core fucosylated structure, as fragments at m/z 474 and 1793 were observed. No fragments showing terminal anntenal fucose residues were detected.

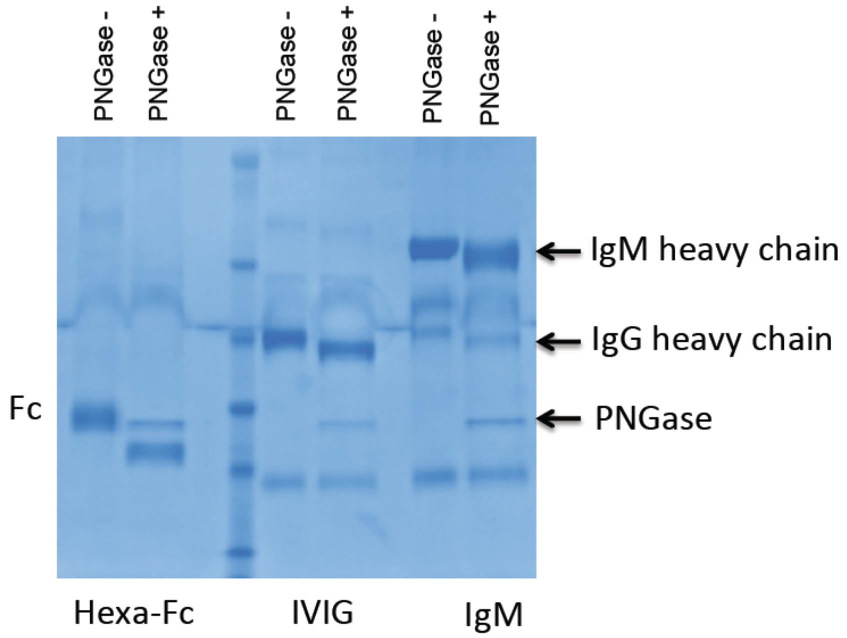

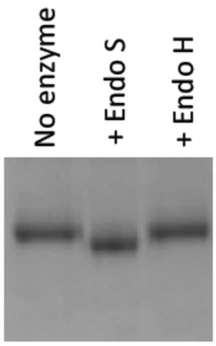

Figure S7. Fifty micrograms of hexa-Fc, human IgM (Sigma) or IVIG (GammaGard) was digested in the presence (+) or absence (-) of PNGase F, Endo S, or Endo H according to manufacturers instructions (New England Biolabs). After overnight incubation at 37°C, 5μg of each antibody preparation was run under reducing conditions into wells of a 4-12% acrylamide gradient gel (Novex) together with Seeblue Plus2 pre-stained protein standards (New England Biolabs).

(A)

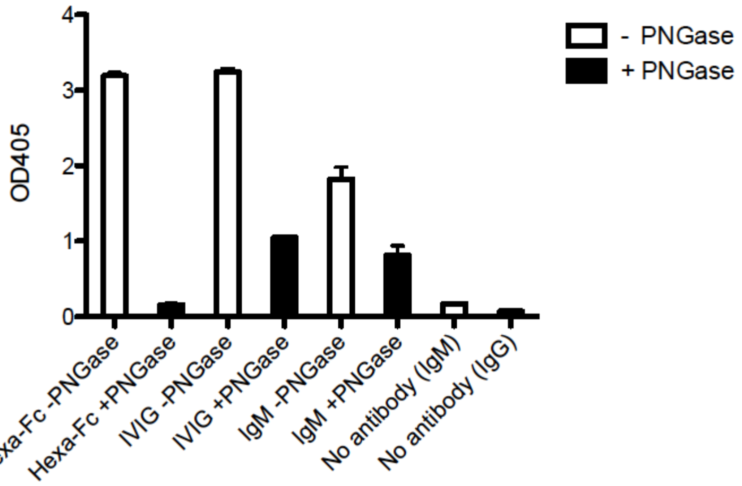

(B)

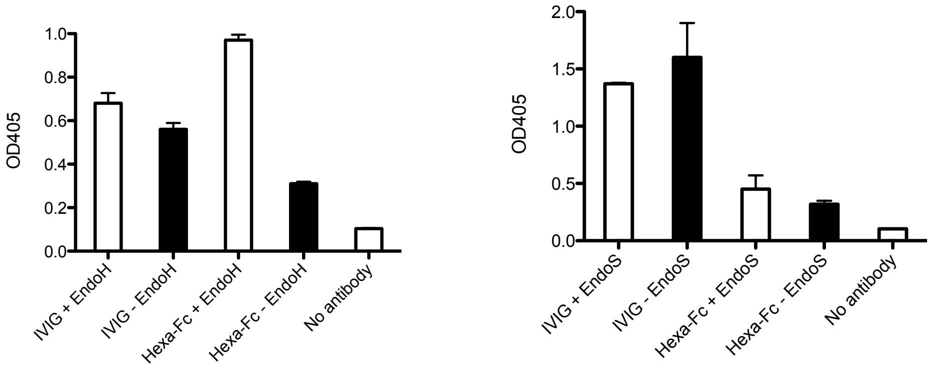

Figure S8. Microtiter wells (Nunc) were coated with DC-SIGN at 10μg/ml in carbonate buffer pH9 and incubated over night at 4°C prior to blocking for 2h at room temperature (RT) in TSM (20mM Tris-HCl, 150mM NaCl, 2mM $CaCl_2$, 2mM $MgCl_2$, 5% BSA) buffer pH 7.4. The wells were washed four times with TSM before addition of 100μl digested or undigested antibodies at 10μg/ml in TBS buffer to duplicate wells. After overnight incubation at 4°C and washing as above, alkaline phosphatase-conjugated anti-human IgG or IgM (1 : 1000; Sigma) was added and incubated for 2h at RT. Wells were washed as above, and 100 μl of the substrate p-nitrophenyl phosphate (Sigma) added to each well and the absorbance measured at 405nm. (A) PNGase F, (B) Endo S or Endo H. Antibodies were treated with enzymes according to manufacturers instructions. Errors bars = SD; n = 2

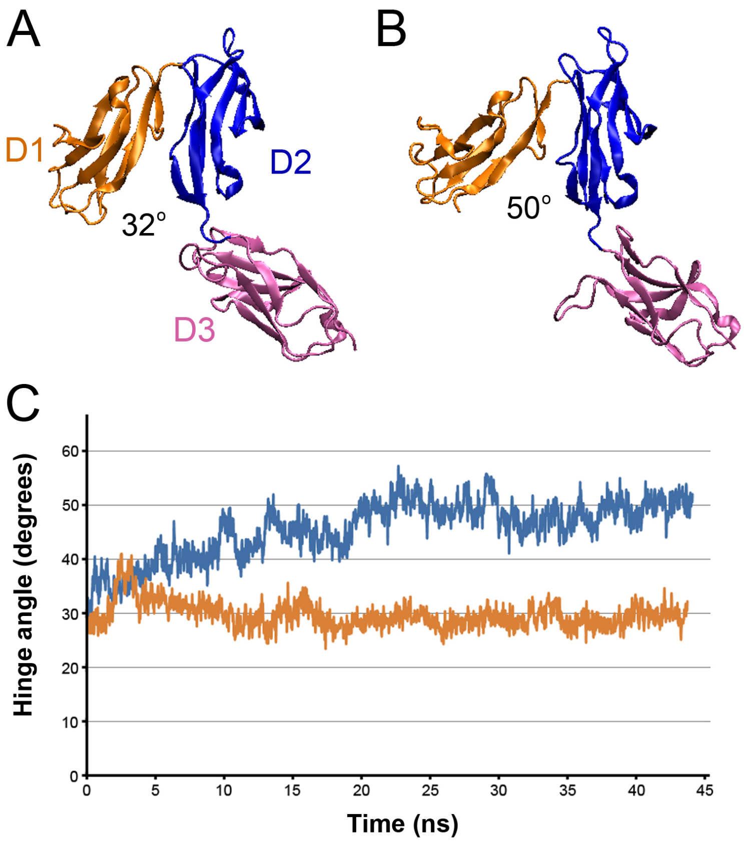

Figure S9. Comparison of the D1-D2 hinge angle in FcRL5 with that in FcγRI. (A) In the initial structure of FcRL5, this angle is ~30º, as it is in the crystal structure of FcγRI, which was used as template for this model. (B) After the structure had equilibrated, this angle in FcRL5 increased to ~50º. (C) The plot shows the magnitude of this hinge angle during the simulations for both FcRL5 (blue) and FcγRI (orange).

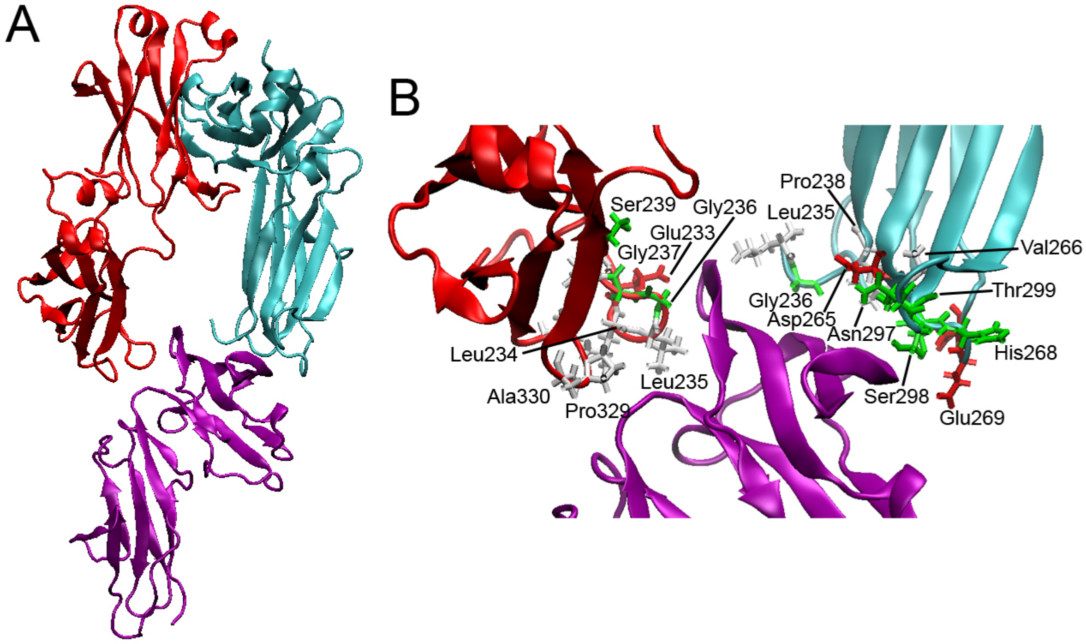

Figure S10. Structural details of the FcγRIII/Fc complex. (A) Overview of the interaction at the end of the simulation. The Fc heavy chains are red and cyan, and FcγRIII is purple. (B) Structural details of the FcγRIII/Fc contact region. Shown as sticks are the Fc residues that are frequently within 3Å of FcγRIII near the end of the simulation to give a sense of the breadth of contact between these two proteins.

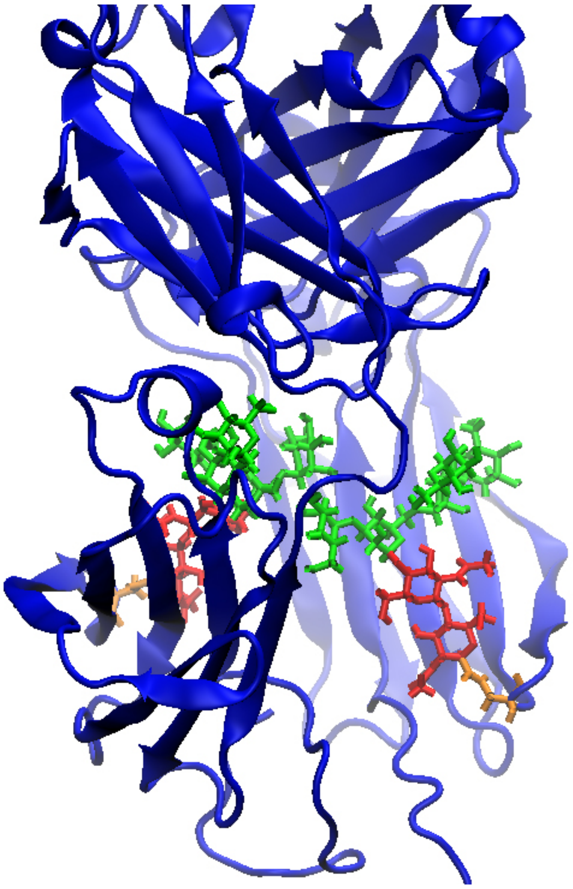

Figure S11. Structural details of the glycans within the Fc domain after ~125ns. The glycans in this structure, and most frequently throughout the trajectory, interact via their α1,3 branch mannose and central β mannose residues. While the α1,6 branch mannose residues in the glycan structure on the left are buried within the cavity, those on the right are clearly located at the cavity entrance. The sugars and protein are coloured as in Figure S1.

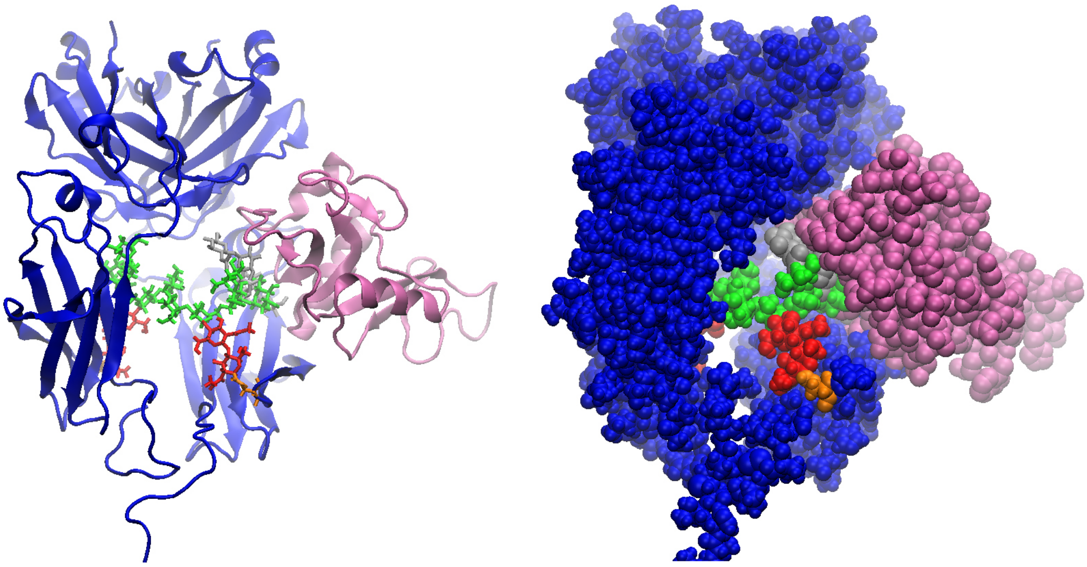

Figure S12. Evaluation of the accessibility of the α1,6 branch mannose residues for the DC-SIGN CRD. The CRD is positioned so that the mannose residues in the crystal structure overlap those in the Fc-glycan. The Fc-glycan is coloured as in Figure S11, the CRD is pink, and the mannose glycan in the CRD crystal structure is grey. The van der Waals representation of the structure (right) more closely reflects the physical structure of the complex.

Supplementary Movie 1.

Trajectory of the Fc/FcRL5 complex during equilibration simulations. The time between adjacent frames is ~0.4ns and this movie reflects a moving average over 3 frames of the original trajectory to enable a clearer appreciation of the relative domain movements. Coloring scheme as in Figure 6.

Supplementary Movie 2.

Trajectory of the Fc/FcγRIII complex. The time between adjacent frames is ~0.4ns and this movie reflects a moving average over 3 frames of the original trajectory. Coloring scheme as in Figure S10.

Supplementary Movie 3.

Trajectory of the glycosylated Fc. The time between adjacent frames is ~0.1ns and this movie reflects a moving average over 3 frames of the original trajectory. The Fc is cyan, the mannose residues are green, and the non-mannose residues are red. Colored orange are the protein residues that are within 3Å of the sugar residues in each frame (roughly hydrogen-binding distance). The glycan in the foreground frequently adopts a configuration in which the mannose residues are near the entrance of the Fc cavity.

Supplementary Movie 4.

Trajectory of the glycosylated Fc. Similar to supplementary movie 3, but with the molecule rotated by 90° about its long axis.
